# Seroprevalence of hepatitis B and C virus among highly active antiretroviral therapy experienced children in Gondar, Ethiopia

**DOI:** 10.1186/s41182-022-00489-2

**Published:** 2022-12-21

**Authors:** Desie Kasew, Mitikie Wondmagegn, Biruk Bayleyegn

**Affiliations:** 1 Department of Medical Microbiology, School of Biomedical and Laboratory Sciences, College of Medicine and Health Sciences, University of Gondar, PO. Box: 196, Gondar, Ethiopia; 2 Department of Medical Microbiology, Debre Tabor University, Debre Tabor, Ethiopia; 3 Department of Hematology and Immunohematology, School of Biomedical and Laboratory Sciences, College of Medicine and Health Sciences, University of Gondar, Gondar, Ethiopia

**Keywords:** Hepatitis, HIV, HAART, Children

## Abstract

**Background:**

Globally, chronic viral hepatitis is the cause of mortality alongside human immunodeficiency virus**/**acquired immunodeficiency syndrome and tuberculosis. Published reports on the seroprevalence of hepatitis B and C viruses among HIV-infected children are lacking in sub-Saharan Africa. Hence, this study aimed to determine the seroprevalence of hepatitis B and C viruses among highly active antiretroviral therapy (HAART)-experienced children at the University of Gondar Comprehensive Specialized Hospital.

**Methods:**

We conducted a hospital-based cross-sectional study to determine the seroprevalence of hepatitis B and C viruses among HAART-experienced children from January to May 2020. We collected the socio-demographic characteristics of study participants with pretested questioners and clinical data from medical records. We performed enzyme-linked immunosorbent assay-based laboratory test for serum hepatitis B surface antigens and anti-hepatitis C virus antibodies. Finally, we analyzed the frequency of all variables, determined the association of independent variables with hepatitis B and C viruses by using univariable and then multivariable logistic regression.

**Results:**

A total of 241 HAART-experienced children were enrolled, 49.8% of whom were girls. The median age of participants was 13 years (interquartile range 11–14). The seroprevalence of hepatitis B and C virus infection among HAART-experienced children were 9.5% and 2.9%, respectively. Being underweight was significantly associated with both hepatitis B virus (AOR = 3.87: 95% CI; 1.04–14.46, *P* = 0.044) and hepatitis C virus infections (AOR = 4.54: 95% CI; 1.21–17.04, *P* = 0.025).

**Conclusions:**

This study showed that the magnitude of hepatitis B and C viruses was high among HIV-infected children who were under HAART and did not know their hepatitis B and C infection status before. Being underweight was associated with both hepatitis viruses. Therefore, screening for hepatitis B and C viruses should be a routine measure for all HIV-infected children.

## Background

Viral hepatitis is a global health issue that claims the lives of 1.34 million people each year. This is more than the human immunodeficiency virus**/**acquired immunodeficiency syndrome (HIV/AIDS) related mortality of 1 million and upcoming to 1.67 million tuberculosis-related mortality in a single year [1–3]. Chronic liver disease associated with hepatitis B virus (HBV) and hepatitis C virus (HCV) is becoming a significant cause of morbidity and mortality among people living with HIV [4]. All three of these viruses share a similar route of transmission [5], such as unsafe sexual contact, blood and blood products, vertical transmission (mainly HBV), horizontal (child-to-child) and injections (mainly HCV) [6] and are preventable; HCV is curable [7].

An estimated 37.7 million people are living with HIV, more than two-thirds of whom dwell in sub-Saharan Africa (SSA) [8]. Among these, 2.7 million are co-infected with HBV and 2.3 million with HCV [9]. According to Barth et al., the rate of HBV infection among people living with HIV (PLWHIV) in Africa is 15%, while HCV infection is 7% [10]. Hepatitis B and C viruses are the common causes of mortality from chronic liver disorders such as Hepatocellular Carcinoma (HCC) and cirrhosis. In SSA, HBV is the common cause of HCC, with 36,700 HCC-related deaths in 2020, and this death rate is expected to double by 2040 [11].

Before the implementation of HAART, coinfection of HIV with HBV and HCV increased mortality [12, 13]. Moreover, HBV infection increases the hepatotoxicity of HAART [14]. Even among HAART-initiated HIV patients, chronic HBV and HCV infections are increasing health challenges [15]. Untreated HIV–hepatitis coinfection, according to the World Health Organization (WHO), hastens progression of HBV and/or HCV–related liver disease, HCC impeding effective HIV treatment [9].

Access to HBV immunization may be lower in HIV-infected infants, or a full dose vaccination may not be protective, and they may be infected by HBV, as a result of a weaker immunologic response and declining titer of antibodies against hepatitis B surface antigens (HBsAg). Besides, the risk of developing chronic hepatitis is about 90% if HBV is acquired during birth or soon after birth, compared to a lower (5%) risk when the infection is acquired in adulthood [16]. Children in SSA are at increased risk of HBV and HCV because home birth is common (> 40%), which increases the risk of perinatal transmission, limits access to HBV vaccination, and needs special attention [17].

In SSA, resources for establishing laboratories with reliable diagnostic tools are limited, which sets back the WHO’s end viral hepatitis target [1]. The prevalence of HBV and HCV infection in HIV-infected children varies significantly with geographic areas and remains to be determined [18]. Those few studies conducted in SSA confirm this, revealing 5.3% in Nigeria and 15% in Tanzania [19, 20]. The Ethiopian Federal Ministry of Health (FMOH) has included HBV vaccination in its Expanded Program of Immunization (EPI) and set a goal of using aggregated national data for evidence-based decisions towards ending viral hepatitis [21]. Published reports on HIV, HBV, and HCV coinfection in children are scarce in the country, even in SSA [22] and lacking in the study area, so determining the local prevalence of these viruses in this segment of population will be the basis for establishing pooled figures in the country. The result will guide health managers, including the Ethiopian FMOH, to give due attention and design effective treatment and control policies. Therefore, this study aimed to provide figures of the seroprevalence and associated factors of HBV and HCV among HAART-experienced children at the University of Gondar Comprehensive Specialized Hospital (UoGCSH).

## Materials and methods

### Study design and area and period

We conducted a cross-sectional study at the UoGCSH ART clinic from January to May 2020 among HAART-experienced HIV-infected children. The hospital is one of the oldest teaching hospitals and is found in the historic town of Gondar, 740 km away from Addis Ababa, the capital city of Ethiopia. The hospital has been providing different health services such as medical, surgical and many other services in both inpatient and outpatient settings for more than 7 million people from Gondar province and neighboring regions. It launched a free-of-charge HIV/AIDS intervention services and has been providing free diagnosis, treatment, and monitoring in its ART clinic for both pediatric and adult patients since 2003.

### Study population

**Fig. 1:**
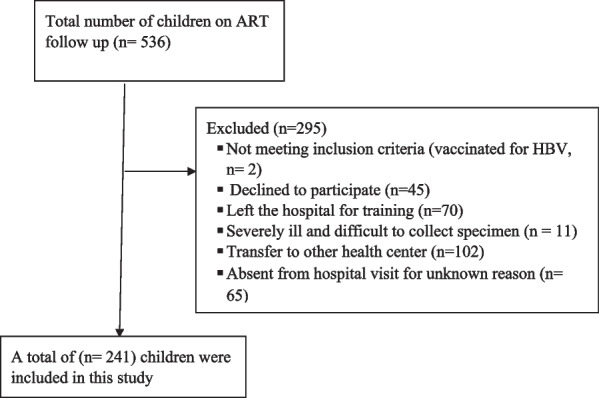
The flow diagram of the inclusion process of study participants

We included 241 HAART-experienced children in this study by using a convenient, non-probabilistic sampling technique. We systematically included all the participants in the study consecutively until the end of the study period (May-2020). This study included all under 15-year-old HIV-infected children attending their follow-up at the UoGCSH, taking HAART and did not know their HBV and HCV status and had no known history HBV vaccination during the study period (Fig. 1).

### Data collection and laboratory procedures

We collected the socio-demographic characteristics of children, including age, gender, residence, educational status, family income, family size, and family occupation by using pretested and structured questioners via a face-to-face interview. Moreover, we gathered the detailed clinical data of the children such as WHO HIV disease stage at the time of sample collection, type of HAART, and duration of HAART they had been taking at the time of sample collection, by reviewing the medical records of HIV-infected children. Based on the WHO guidelines, the most commonly prescribed HAART regimen consists of nucleoside reverse transcriptase inhibitors (NRTIs: lamivudine (3TC), abacavir (ABC), and zidovudine (AZT), tenofovir disoproxil fumarate (TDF)) together with a non-nucleoside reverse transcription inhibitor (NNRTI, nevirapine or efavirenz) or a protease inhibitor (lopinavir). Currently, the preferred HAART regimen for those children is the release of a new, dispersible tablet formulation of dolutegravir (DTG) plus two nucleoside reverse transcriptase inhibitors (NRTIs) [23]. As the study participants were HIV-infected children, WHO anthro (for children aged ≤ 5 years) and anthro-plus (for children aged > 5 years) software were applied for calculation of their anthropometric measures such as *Z*-scores of Weight-for-Age (WAZ), Height-for-Age (HAZ) and Body Mass Index for Age (BAZ) based on WHO nutritional assessment guideline. Underweight children are those whose *z*-score of weight-for-age (WAZ) is less than − 2, while stunted means children who have lower than height-for-age of less than − 2 *Z*-scores [24].

Regarding the laboratory method, 5 ml of venous blood was collected following standard operating procedures (SOPs) for HIV-viral load determination, centrifuged to extract serum for the analysis of HBsAg and anti-HCV antibodies using enzyme-linked immunosorbent assay (ELISA). The serum specimen was stored at – 80 °C until processed and brought to room temperature and analyzed by thawing once before analysis. Then, the HBsAg and anti-HCV antibody (IgG and IgM) were detected on sera using ELISA according to the manufacturer’s instructions using AiD™ HBsAg ELISA and Wantai Aid™ anti-HCV ELISA ^Plus^, ELISA kits (Beijing Wantai Biological Pharmacy Enterprise Co., Ltd, China). Furthermore, HIV Ribonucleic acid (RNA) viral load was determined directly by an advanced molecular technique using TAQMAN® AMPLICOR HIV-1 MONITOR (Roche Molecular Systems) according to the manufacturer's instruction by well-trained laboratory technologists. To ensure the quality of data, pre-analytical, analytical, and post-analytical phases were strictly maintained. Safety procedures and specimen handling procedures were strictly followed for the tests. (ELIZA, and HIV viral load). The manufacturer procedures and SOPs were strictly followed.

### Data analysis and interpretation

Data were coded and entered into EPI-info version 4.4 and were transferred to Statistical Package for Social Science (SPSS) version 20 for analysis. Descriptive statics like frequencies and percentages were used to summarize the results. The distribution of data was assessed through Shapiro–Wilk test and a *p*-value > 0.05 in the Shapiro–Wilk test was considered as normally distributed data. Both crude odds ratio (COR) and adjusted odds ratio (AOR) with 95% confidence interval (CI) were calculated to determine the extent to which the risk factors are associated with hepatitis infection. Variables such as sex, age, WAZ grouped, BAZ grouped, HAZ grouped, educational status, family marital status, family educational status, family size, lymphopenia, anemia, WHO HIV stage, HIV viral load were subjected to univariable analysis for calculating COR. To identify the independent factors, variables with *p* < 0.2 at univariable analysis were included in multivariable analysis. The model was built by dropping the most insignificant factors one at a time in a stepwise manner and finally, in the multivariable analysis, a variable with a *p*-value < 0.05 was considered as statistically significant.

## Results

### Socio-demographic and clinical characteristics of HAART-experienced children

The total number of study participants in the study was 241, of whom 121 (50.2%) were boys. Among these study participants, 86.7% had been taking the HAART for a year or longer. The median age of participants was 13 years, with an interquartile range (IQR)-of 11–14. In addition, 232 (96.4%), 52 (21.6%), and 204 (84.6%) of the participants were in the WHO clinical stage of HIV-I, had an HIV viral load of above 1000 (both at the time of data collection), and had been living with HIV-infected parents, respectively. All 241 of the study participants were on ART regimens that contain 3TC in its backbone. The overall seroprevalence of HBsAg and anti-HCV Abs among HAART-experienced children who did not know their HBV and/or HCV infection status before, was 12.4% (95% CI = 8.7–17). The seroprevalence of HBsAg was 9.5% (*N* = 23, 95%CI: 6.6–13.2), and anti-HCV Abs were 2.9% (*N* = 7, 95% CI = 0.8–5.8). There was no coinfection of HBV and HCV in this study (Table 1).

**Table 1:** Socio-demographic and clinical characteristics of HAART experienced children at the UoGCSH, 2020

Variables	Category	*N* (%)
Sex	Male	121 (50.2)
	Female	120 (49.8)
Age	2–10	59 (24.5)
	11–15	182 (75.5)
Residence	Urban	215 (89.2)
	Rural	26 (10.8)
Education	No	20 (8.3)
	Primary	191 (79.3)
	Secondary	30(12.4)
Marital status of family	Married	129 (53.5)
	Separate/single	30 (12.4)
	Widowed	82 (34)
Family monthly income (ETB)	< 1000	117 (48.5)
	1000–2000	72 (29.9)
	> 2000	52 (21.6)
Family size	2–4	172 (71.4)
	5–8	69 (28.6)
HIV status of care giver	Positive	204 (84.6)
	Negative	37 (15.4)
HIV Viral load (at the time of data collection)	Not detected	112 (46.5)
	≤ 1000	77 (32)
	> 1000	52 (21.6)
WHO HIV stage (at the time of data collection)	I	232 (96.4)
	II	9 (3.7)
HAART classification	ABC-3TC-LPV/r	19 (7.9)
	ABC-3TC-DTG	46 (19.1)
	ABC-3TC-NVP	6(2.5)
	AZT-3TC-EFV	26(10.8)
	AZT-3TC-LPV/r	7(2.9)
	AZT-3TC-NVP	39(16.2)
	TDF-3TC-DTG	50(20.7)
	TDF-3TC-EFV	26(10.8)
	TDF-3TC-LPV/r	14(5.8)
	Others	8(3.3)
WAZ group	Under weight	15 (6.2)
	Normal	226 (93.8)
HAZ group	Stunted	106 (44)
	Normal	135 (56)
BAZ group	Under weight	41 (17)
	Normal	200 (83)
Hepatitis seropositivity	HBsAg positive	23 (9.5)
	Anti-HCV Abs positive	7 (2.9)

Others: ABC-3TC-EFV = 3, AZT-3TC-DTG = 3, and TDF-3TC-NVP = 2, 3TC- lamivudine, ABC: abacavir, AZT: zidovudine, DTG: dolutegravir, EFV: efavirenz, LPV/r: lopinavir/ritonavir, NVP: nevirapine, TDF: tenofovir disoproxil fumarate, ETB: Ethiopian birr, WAZ: Weight-for-Age, HAZ: Height-for-Age, BAZ: Body Mass Index for Age

### The association of socio-demographic features of HAART-experienced children with HBV infection

Among the variables computed in univariable logistic regression to determine the association with HBsAg seropositivity, weight (WAZ) (COR = 3.96: 95% CI; 1.15–13.65 and *P* = 0.029) and HIV-viral load (COR = 3.14: 95% CI; 1.10–8.97 and *P* = 0.033) were entered to and analyzed in multivariable analysis. The result of multivariable analysis revealed that WAZ was significantly associated with HBsAg seropositivity (AOR = 3.87: 95% CI; 1.04–14.46, and *P* = 0.044) (Table 2).

**Table 2:** Factors associated with HBV infection among HAART experienced children at the UoGCSH, 2020

Variables	Category	HBV infection		COR (95% CI)	*P*-value	AOR (95% CI)	*P*-value
		Yes *N* (%)	No *N* (%)				
Sex	Female	11(9.2)	109(90.8)	1			
	Male	12(9.9)	109(90.1)	1.09(0.46–2.58)	0.843		
Age	2–10	4(6.8)	55(93.2)	1			
	11–15	19(10.4)	163(89.6)	1.60(0.52–4.92)	0.409		
WAZ grouped	Normal	19(8.4)	207(91.6)	1		1	
	Under weight	4(26.7)	11(73.3)	3.96(1.15–13.65)	0.029*	3.87(1.04–14.46)	0.044*
BAZ grouped	Normal	19(9.5)	181(90.5)	1			
	Under weight	4(9.8)	37(90.2)	1.03(0.33–3.20)	0.960		
HAZ grouped	Normal	13(9.6)	122(90.4)	1			
	Stunted	10(9.4)	96(90.6)	0.98(0.41–2.33)	0.960		
Educational status	Secondary	1(3.3)	29(96.7)	1			
	Primary	21(11)	170(89)	3.58(0.46–27.67)	0.221		
	No formal education	1(5)	19(95)	1.53(0.09–25.90)	0.770		
Family size	2–4	14(8.1)	158(91.9)	1			
	5–8	9(13)	60(87)	1.69(0.69–4.12)	0.246		
Family educational status	No formal	7(8.2)	78(91.8)	1			
	Primary	8(10.3)	70(89.7)	1.27(0.44–3.69)	0.656		
	Secondary and above	8(10.3)	70(89.7)	1.27(0.44–3.69)	0.656		
Marital status of family	Married	11(8.5)	118(91.5)	1			
	Single	3(10)	27(90)	1.19(0.31–4.57)	0.789		
	Widowed	9(11)	73(89)	1.32(0.52–3.35)	0.555		
WHO HIV stage (at the time of data collection)	I	22(9.5)	210(90.5)	1			
	II	1(11.1)	8(88.9)	1.19(0.14–9.99)	0.871		
Viral Load rate (at the time of data collection)	Not detected	7(6.2)	105(93.8)	1		1	
	≤ 1000	7(9.1)	70(90.9)	1.50(0.50–4.46)	0.466	1.52(0.50–4.56)	0.460
	> 1000	9(17.3)	43(82.7)	3.14(1.10–8.97)	0.033	2.80(0.95–8.24)	0.062

*Significantly associated

### The association of socio-demographic and clinical features of HAART-experienced children with HCV infection

Regarding the association variables with anti-HCV seropositivity, the variable WAZ showed a statistically significant association with HCV infection (AOR = 4.54: 95% CI; 1.21–17.04 and *P* = 0.025) (Table 3).

**Table 3:** Factors associated with HCV infection among HAART-experienced children at the UoGCSH, 2020

Variables	Category	HCV infection		COR (95% CI)	*P*-value	AOR (95% CI)	*P*-value
		Yes *N* (%)	No *N* (%)				
Sex	Female	4(3.3)	116(96.7)	1			
	Male	3(2.5)	118(97.5)	0.74(0.16–3.37)	0.694		
Age	2–10	2(3.4)	57(96.6)	1			
	11–15	5(2.7)	177(97.3)	0.81(0.15–4.26)	0.799		
WAZ grouped	Normal	5(2.2)	221(97.8)	1		1	
	Under weight	2(13.1)	13(86.7)	6.80(1.20–38.46)	0.030*	4.54(1.21–17.04)	0.025
BAZ grouped	Normal	5(2.5)	195(97.5)	1			
	Under weight	2(4.9)	39(95.1)	2.00(0.37–10.68)	0.417		
HAZ grouped	Normal	3(2.2)	132(97.8)	1			
	Stunted	4(3.8)	102(96.2)	1.73(0.38–7.88)	0.482		
Educational status	Secondary	1(3.3)	29(96.7)	1		1	
	Primary	3(1.6)	188(98.4)	0.46(0.04–4.60)	0.511	3.69(0.45–30.10)	0.223
	No formal education	3(15)	17(85)	5.12(0.49–53.18)	0.172	2.99(0.16–55.70)	0.464
Family marital status	Married	1(0.8)	128(99.2)	1		1	
	Single	2(6.7)	28(93.3)	9.14(0.81–104.38)	0.075	1.18(0.29–4.78)	0.819
	Widowed	4(4.9)	78(95.1)	6.56(0.72–59.800)	0.095	1.55(0.56–4.32)	0.400
Family educational status	Secondary and above	1(1.3)	77(98.7)	1		1	
	Primary	5(6.4)	73(93.6)	5.27(0.60–46.23)	0.133	1.02(0.34–3.05)	0.969
	No formal education	1(1.2)	84(98.8)	0.92(0.06–14.91)	0.951	0.76(0.23–2.49)	0.649
Family size	2–4	6(3.5)	166(96.5)	1			
	5–8	1(1.4)	68(98.6)	0.407(0.05–3.44)	0.409		
Viral load rate (at the time of data collection)	Not detected	2(1.8)	110(98.2)	1			
	≤ 1000	3(3.9)	74(96.1)	2.23(0.36–13.67)	0.386		
	> 1000	2(3.8)	50(96.2)	2.20(0.30–16.07)	0.437		

## Discussion

The World Health Organization designed a strategy for eliminating viral hepatitis as part of the Global Health Sector Strategy (GHSS) agenda 2030 for sustainable development. It aimed to succeed in diagnosing 90% of infected individuals, treating 80% of diagnosed cases, reducing death by 65%, and vaccinating 90%. Unfortunately, less than 5% of chronic viral hepatitis patients know their status. Knowledge of the burden of HBV and HCV and incorporation of the target population who needs the services in each country are two of the five strategic directions for the GHSS goal [1]. Therefore, this study reveals the seroprevalence of HBV and HCV among HIV-infected children (the population who need the services), which supports determining the national prevalence of viral hepatitis in this population segment.

All the study participants received ART containing 3TC in the backbone as the Ethiopian HIV treatment guidelines recommended. Lamivudine (3TC) is also active against HBV and is commonly used in the treatment of HBV or combined with other drugs for the treatment of HIV or HIV–HBV [25]. The 3TC is accessible at a lower cost in resource-limited settings like Ethiopia [26]. The national HIV treatment guideline requests screening for baseline HBV serology (HBsAg) [25] But HBV diagnosis before initiation of HIV treatment and close monitoring of patients on for HBV viremia and biochemical status are not implemented in Ethiopia. Mutation and rapid progression of drug resistance, exacerbation of HBV disease and liver enzyme flare are the potential risks in this high HIV/HBV burden area [26, 27]. This study did not assess the effect of 3TC because all the study participants were exposed to the drug, tested once and not followed for HBV response.

The overall seropositivity rate of hepatitis among HAART-experienced children was 12.4% (95% CI = 8.7–17). From these, 9.5% were seropositive for HBsAg and the rest 2.9% were seropositive for anti-HCV antibodies. The overall result was comparable to the seropositivity rate of both HBsAg and anti-HCV reported in Nigeria (14.0%) [28]. The result was also analogous to results reported in Guinea, (HBsAg = 8.16%) [29], Sierra Leone (HBsAg = 7.4% and anti-HCV = 1.5%) among children receiving ART[30];Tanzania (HBsAg = 7%) [31], Rwanda (HBsAg = 7%) [32], and Nigeria (HBsAg = 10% and anti-HCV = 1.7%) among ART-naïve HIV-infected children [33]. The SSA is a region of high HBV endemicity (8% and above) [34]. The result of our study indicates that the seroprevalence of HBV and HCV is high among children who are taking HAART.

The result of this study reveals a higher positivity rates of HBsAg compared to results reported by investigators from West Africa (2.2–5.3%) [20, 35–38] and comparable positivity rates of anti-HCV (0.5–3.9%) results from the same region [20, 37, 39].

The variation might be because of population and geographic differences. Some authors included infants, while others included those adolescents who were aged above 15 years old. But, in this study, children aged 2 to 15 years old were the study population. Efforts to ensure health care, expand HBV vaccination programs, and the goal of ending viral hepatitis might also be responsible for the difference. The result of this study indicates that Ethiopia is far behind the goal set by the WHO to end viral hepatitis by 2030 [40]. The WHO recommendations for routine screening of HIV patients for HBV and HCV infections were not implemented in Ethiopia, in which resources are limited, and the result of our study reflects this weak move towards the target [41].

Unlike the result of this study, a study involving ART-naïve HIV-infected children in Bahir Dar, Ethiopia, reported a reduced seroprevalence of HBV (HBsAg = 2%) and a borderline higher HCV (anti-HCV = 5.5%) [18]. In addition, a study done in Nigeria reported a lower HBV (HBsAg = 3%) and a higher HCV (anti-HCV = 11%) infection among HAART-experienced children [28]. The difference in the length of exposure to HAART and anti-HBV activity of the regimens could have reduced HBV infection. In contrast, the higher HCV infection rate might be attributed to weak immune reconstitution in these children. Hepatitis C virus coinfection in HAART-experienced HIV patients has been shown to have a low immune/CD4 reconstitution [18, 41].

Among the variables analyzed in this study, weight was the variable significantly associated with HBV and HCV infections. The likelihood of HBV seropositivity in HAART-experienced underweight children was nearly fourfold higher than the likelihood of HBV seropositivity in normal-weight children (AOR = 3.87). Similarly, the odds of HCV seropositivity among underweight (WAZ) children was at least 4.5 times higher than the odds of HCV among normal-weight children. The higher likelihood of hepatitis seropositivity among underweight children might be associated with poor nutritional status and associated poor immune status or immunosuppression by HIV infection, and the resulting lower clearance capacity for the viruses. Strengths of this research: this research focused on the high-risk population segment. Hence, it can be an example of the local prevalence of HBV and HCV among HAART experienced children living with HIV in the high burdened SSA region. We included large sample size and ELISA rather than rapid diagnostic techniques for detection. It can also be used as baseline data for further study and control strategy. This study was limited by the lack of data on the history of vaccination, absence of HBV-core antibody tests and molecular tests for detection HBV and HCV, lack of longitudinal data and absence of liver function tests. There were two children who had history of vaccination and were excluded.

## Conclusions

The findings of this study showed that the seroprevalence of hepatitis B and C virus infection was high among HAART-experienced children with unknown previous hepatitis status. The variable being underweight was associated with both hepatitis B and C viruses. Therefore, screening HIV-infected children and implementing appropriate remedial measures are crucial to reducing these infections. Large-scale population-based studies on are needed to determine the national prevalence to help reach the WHO end viral hepatitis goal by 2030.

## Data Availability

All data generated and analyzed during this study were included in this article. Data supporting the findings of this study are also available at the corresponding author for a reasonable request.

## Abbreviations

AOR: Adjusted odds ratio
ART: Antiretroviral therapy
BAZ: Body Mass Index for Age Z-score
CI: Confidence interval
COR: Crude odds ratio
ELISA: Enzyme linked immunosorbent assay
HAART: Highly active antiretroviral therapy
HAZ: Height-for-Age Z-score
HBsAg: Hepatitis B surface antigen
HBV: Hepatitis B virus
HCC: Hepatocellular carcinoma
HCV: Hepatitis C virus
HIV/AIDS: Human immunodeficiency virus/acquired immunodeficiency syndrome
PLWHIV: People living with HIV
SSA: Sub-Saharan Africa
UoGCSH: University of Gondar Comprehensive Specialized Hospital
WAZ: Weight-for-Age Z-score
WHO GHSS: World Health Organization Global Health Sector Strategy

## References

[1] World Health Organization. Global health sector strategy on viral hepatitis 2016–2021. Towards ending viral hepatitis. World Health Organization, 2016.

[2] Unaids J (2017). Fact sheet—latest global and regional statistics on the status of the AIDS epidemic.

[3] World Health Organization. Global hepatitis report 2017: World Health Organization; 2017.

[4] Rosenthal E, Roussillon C, Salmon-Céron D, Georget A, Hénard S, Huleux T (2015). Liver-related deaths in HIV-infected patients between 1995 and 2010 in France: the Mortavic 2010 study in collaboration with the Agence Nationale de Recherche sur le SIDA (ANRS) EN 20 M ortalité 2010 survey. HIV Med.

[5] Vickers NJ (2017). Animal communication: when i’m calling you, will you answer too?. Curr Biol.

[6] World Health Organization. Management of hepatitis B and HIV coinfection, Clinical Protocol for the WHO European Region 2011 revision. 2011.

[7] Singh PK (2018). Towards ending viral hepatitis as a public health threat: translating new momentum into concrete results in South-East Asia. Gut Pathogens.

[8] World Health Organization. HIV/AIDS _Fact sheet 2020 https://www.who.int/news-room/fact-sheets/detail/hiv-aids. Accessed 2020.

[9] World Health Organization. Global progress report on HIV, viral hepatitis and sexually transmitted infections, 2021: accountability for the global health sector strategies 2016–2021: actions for impact: web annex 2: data methods. 2021.

[10] Barth RE, Huijgen Q, Taljaard J, Hoepelman AI (2010). Hepatitis B/C and HIV in sub-Saharan Africa: an association between highly prevalent infectious diseases. A systematic review and meta-analysis. Int J Infect Dis.

[11] Amponsah-Dacosta E (2021). Hepatitis B virus infection and hepatocellular carcinoma in sub-Saharan Africa: implications for elimination of viral hepatitis by 2030?. World J Gastroenterol.

[12] Miailhes P, Trabaud M-A, Pradat P, Lebouché B, Chevallier ML, Chevallier P (2007). Impact of highly active antiretroviral therapy (HAART) on the natural history of hepatitis B virus (HBV) and HIV coinfection: relationship between prolonged efficacy of HAART and HBV surface and early antigen seroconversion. Clin Infect Dis.

[13] Sadoh A, Sadoh W, Iduoriyekemwen N (2011). HIV co-infection with hepatitis B and C viruses among Nigerian children in an antiretroviral treatment programme. S Afr J Child Health.

[14] Ayuk J (2013). Hepatitis B virus in HIV-infected patients in northeastern South Africa: prevalence, exposure, protection and response to HAART. S Afr Med J.

[15] Taylor LE, Swan T, Mayer KH (2012). HIV coinfection with hepatitis C virus: evolving epidemiology and treatment paradigms. Clin Infect Dis.

[16] Healy SA, Gupta S, Melvin AJ (2013). HIV/HBV coinfection in children and antiviral therapy. Expert Rev Anti Infect Ther.

[17] World Health Organization. Prevention of mother-to-child transmission of hepatitis B virus: guidelines on antiviral prophylaxis in pregnancy: web annex A: systematic review of the efficacy and safety of antiviral therapy during pregnancy. 2020. 32833415

[18] Abera B, Zenebe Y, Mulu W, Kibret M, Kahsu G (2014). Seroprevalence of hepatitis B and C viruses and risk factors in HIV infected children at the Felgehiwot referral hospital, Ethiopia. BMC Res Notes.

[19] Telatela SP, Matee MI, Munubhi EK (2007). Seroprevalence of hepatitis B and C viral co-infections among children infected with human immunodeficiency virus attending the paediatric HIV care and treatment Center at Muhimbili National Hospital in Dar-es-Salaam, Tanzania. BMC Public Health.

[20] Lawal MA, Adeniyi OF, Akintan PE, Salako AO, Omotosho OS, Temiye EO (2020). Prevalence of and risk factors for hepatitis B and C viral co-infections in HIV infected children in Lagos, Nigeria. PLoS ONE.

[21] Ministry of Health Ethiopia. National Strategy for Prevention and Control of Viral Hepatitis 2016–2020. 2016.

[22] Tamandjou Tchuem CR, Brandt L, Nel EDLR, Cotton MF, Matthews P, Kaindjee-Tjituka F (2020). Hepatitis B virus drug resistance mutations in HIV/HBV co-infected children in Windhoek, Namibia. PLoS ONE.

[23] World Health Organization. Panel on Antiretroviral Therapy and Medical Management of Children Living with HIV. Guidelines for the use of antiretroviral agents in pediatric HIV infection. 2021.

[24] World Health Organization (2013). Guideline: updates on the management of severe acute malnutrition in infants and children.

[25] Ministry of health Ethiopia. National consolidated guidelines for comprehensive HIV prevention. Care and Treatment. 2018.

[26] Ocama P, Seremba E, Apica B, Opio K (2015). Hepatitis B and HIV co-infection is still treated using lamivudine-only antiretroviral therapy combination in Uganda. Afr Health Sci.

[27] Telele NF, Kalu AW, Gebre-Selassie S, Fekade D, Marrone G, Grossmann S (2019). A viral genome wide association study and genotypic resistance testing in patients failing first line antiretroviral therapy in the first large countrywide Ethiopian HIV cohort. BMC Infect Dis.

[28] Pennap G, Yahuza A, Abdulkarim M, Oti V (2016). Prevalence of Hepatitis B and C viruses among human immunodeficiency virus infected children attending an antiretroviral therapy clinic in Lafia, Nigeria. Asia J Appl Microbiol.

[29] Kaba D, Bangoura MA, Sylla MM, Sako FB, Diallo MS, Diallo I (2019). Prevalence and factors associated with hepatitis B in a cohort of HIV-infected children in the Pediatric Department at Donka National Hospital, Guinea. Pan Afr Med J.

[30] Yendewa GA, Lakoh S, Yendewa SA, Bangura K, Lawrence H, Patiño L (2021). Prevalence of hepatitis B surface antigen and serological markers of other endemic infections in HIV-infected children, adolescents and pregnant women in Sierra Leone: a cross-sectional study. Int J Infect Dis.

[31] Muro FJ, Fiorillo SP, Sakasaka P, Odhiambo C, Reddy EA, Cunningham CK (2013). Seroprevalence of hepatitis B and C viruses among children in Kilimanjaro Region, Tanzania. J Pediatr Infect Dis Soc.

[32] Mutwa PR, Boer KR, Rusine JB, Muganga N, Tuyishimire D, Reiss P (2013). Hepatitis B virus prevalence and vaccine response in HIV-infected children and adolescents on combination antiretroviral therapy in Kigali, Rwanda. Pediatr Infect Dis J.

[33] Durowaye M, Ernest S, Ojuawo I (2014). Prevalence of HIV co-infection with Hepatitis B and C viruses among children at a tertiary hospital in Ilorin, Nigeria. Int J Clin Med Res.

[34] Weldemhret L (2021). Epidemiology and challenges of HBV/HIV co-infection amongst HIV-infected patients in endemic areas. HIV/AIDS (Auckland, NZ).

[35] Varo R, Chris Buck W, Kazembe PN, Phiri S, Andrianarimanana D, Weigel R (2016). Seroprevalence of CMV, HSV-2 and HBV among HIV-infected Malawian children: a cross-sectional survey. J Trop Pediatr.

[36] Joseph F, Rodrigue KW, Serges T, Salomon NP, Christian TN, Carlos TTM (2019). Hepatitis B infection and risk factors among children living with HIV in Yaounde, Cameroon: an integrated management. BMC Pediatr.

[37] Okechukwu A, Thairu Y, Dalili M (2020). HIV co-infection with hepatitis B and C and liver function in children and adolescents on antiretroviral therapy in a tertiary health institution in Abuja. West Afr J Med.

[38] Toyé RM, Lô G, Diop-Ndiaye H, Cissé AM, Ndiaye AJS, Kébé-Fall K (2021). Prevalence and molecular characterization of hepatitis B virus infection in HIV-infected children in Senegal. Clin Res Hepatol Gastroenterol.

[39] Onyire NB, Orji ML, Ogah EO (2017). Prevalence of Hepatitis B and C Infections in children infected with human immunodeficiency virus in Abakaliki, Ebonyi State, Southeast, Nigeria. Malay J Paediatr Child Health..

[40] World Health Organization. End hepatitis by 2030: prevention, care and treatment of viral hepatitis in the African Region: framework for action, 2016–2020. 2017.

[41] World Health Organization. Antiretroviral therapy of HIV infection in infants and children: towards universal access: recommendations for a public health approach-2010 revision. World Health Organization. 2010. 23741772

